# Case Report: Semi-Ex Vivo Hepatectomy Combined with Autologous Liver Transplantation for Alveolar Echinococcosis in Children

**DOI:** 10.4269/ajtmh.23-0276

**Published:** 2023-08-07

**Authors:** Pan Xia, Xiang-Qian Wang, Qing-Shan Tian, Chao-Liang Shang-Guan, Hai-Hong Zhu

**Affiliations:** ^1^ Department of Graduate School, Qinghai University, Xining, China;; ^2^ Department of General Surgery, Qinghai Provincial People’s Hospital, Xining, China

## Abstract

Hepatic alveolar echinococcosis (AE) is a zoonotic disease caused by the metacestode of *Echinococcus multilocularis*. Although surgical resection is the optimal treatment for hepatic AE, some patients with hepatic AE located in special introhepatic sites cannot be radically cured by conventional surgery. Here, we report that a 10-year-old female patient was admitted to the hospital with occupying liver lesions for 6 months. Computed tomography examination showed irregular mixed-density masses in the right lobe and caudate lobe of the liver, with partial invasion of the right hepatic artery, right hepatic vein, and right branch of the portal vein. The patient was preoperatively diagnosed with hepatic AE, which cannot be cured by conventional liver lobectomy. The patient underwent semi-ex vivo liver resection with autologous liver transplantation (second hepatic portal reconstruction, posterior hepatic inferior vena cava repair, and hepatic artery repair) and biliary-intestinal anastomosis. After hospital discharge, she has kept living a healthy life without disease recurrence for 13 months until the end of the last follow-up. This case shows that semi-ex vivo hepatectomy with autologous liver transplantation might be a feasible and safe choice for certain patients with AE located in special introhepatic sites, which has provided novel experiences for the surgical treatment of hepatic AE.

## INTRODUCTION

Hepatic alveolar echinococcosis (AE) is a zoonotic disease caused by the metacestode of *Echinococcus multilocularis*, which is distributed in pastoral areas of all continents in the world.^1^ Hepatic AE is a serious disease, with high pathogenicity, disability, and mortality rates. The 10-year mortality rate of AE patients without effective treatment is 94%.^2^ Moreover, hepatic AE is most dangerous for children, with some experts estimating that 60 million people are currently at risk of infection worldwide, with approximately 2–3 million cases of echinococcosis, one-third of which occur in children.^3^ The preferred treatment of hepatic AE is radical surgical resection. However, for some patients with hepatic AE in special sites, it cannot be removed by conventional radical surgery. Therefore, some unconventional methods have been used to solve this complex situation.^4^ This case reports a female pediatric patient with hepatic AE who underwent semi-ex vivo hepatectomy with autologous liver transplantation because radical resection was not possible with conventional surgical approaches, providing experiences in the treatment modalities for unresectable hepatic AE in children.

## CASE REPORT

A 10-year-old girl with a height of 1.32 m and a weight of 26.0 kg was admitted to the Qinghai Provincial People’s Hospital on February 25, 2022, with a 6-month history of hepatic occupying lesions. Because the patient did not have any uncomfortable symptoms, she did not visit the hospital earlier. After a reexamination at the local hospital, the patient was advised to come to our hospital for further treatment. No abnormal physical examination was detected at the time of admission.

**Figure 1.**
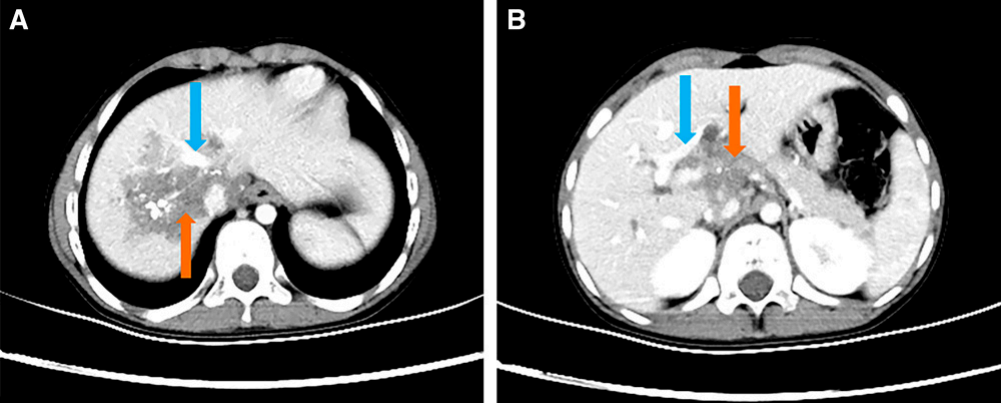
(**A**) Hepatic SVII and SVIII alveolar echinococcosis lesions (light-colored arrow) and the middle hepatic vein (dark arrow). (**B**) Hepatic SI and SIV alveolar echinococcosis lesions (light-colored arrow) and the hepatic portal vein (dark arrow).

**Figure 2.**
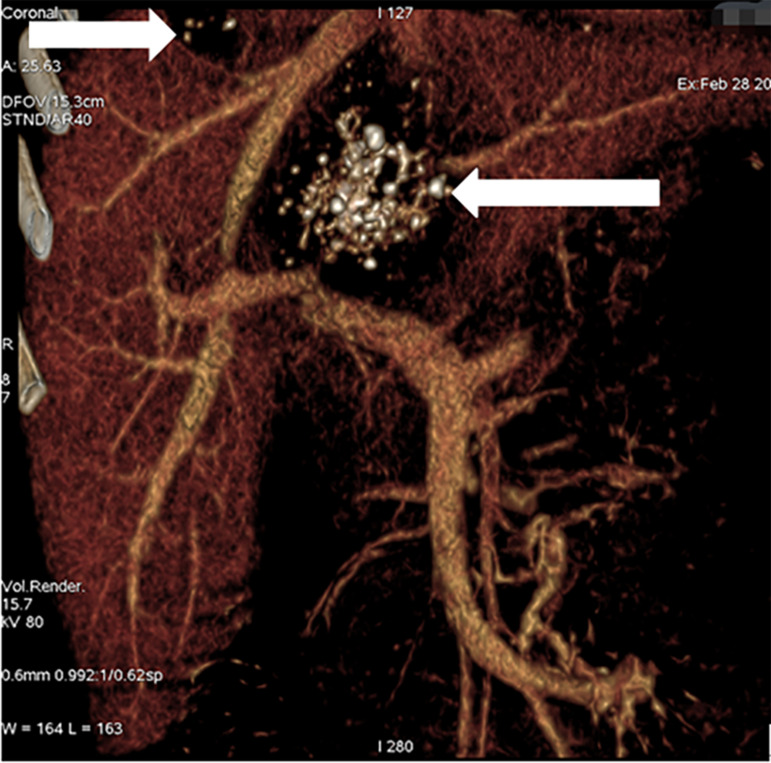
Alveolar echinococcosis lesions (white arrows).

The patient’s laboratory test results are shown in Table 1. On the basis of laboratory tests, the patient was diagnosed with hepatitis B before surgery and received antiviral treatment. Computed tomography (CT) examination showed irregular mixed-density masses in the right lobe and caudate lobe of the liver with a large cross-section of approximately 57.8 × 50.0 mm in which punctate calcification was seen, with no enhancement on the enhanced scan (Figure 1A and B). CT angiography of the hepatic artery, inferior vena cava, and portal vessels showed a mixed-density mass in the right lobe of the liver and caudate lobe, with partial invasion of the right hepatic artery, right hepatic vein, and right branch of the portal vein (Figure 2). On the basis of these imaging findings, the patient was preoperatively diagnosed with hepatic AE (invasion of the first and second hepatic portal and inferior vena cava).

**Table 1:** The patient’s laboratory test results

Laboratory index	Results	Reference value
WBC	4.41	3.50–9.50 × 10^9^/L
EO%	8.8%	0.4–8.0%
ALT	64 U/L	7–45 U/L
AST	81 U/L	13–40 U/L
GGT	132 U/L	7–45 U/L
TBIL	12.3 μmol/L	5.0–21.0 μmol/L
ALB	35.1 g/L	40–55 g/L
PT	10.6 seconds	10.0–14.0 seconds
HBsAg	77124.95 IU/mL	< 0.05 IU/mL
HBV-DNA	8.69 × 10^7^ IU/mL	< 30 IU/mL
ELISA	4.52	< 0.9
R15	1.4%	< 10%

ALB = albumin; ALT = alanine aminotransferase; AST = aspartate aminotransferase; EO% = percentage of eosinophils; GGT = gamma glutamyltransferase; HBsAg = hepatitis B surface antigen; HBV-DNA = HBV DNA content; PT = prothrombin time; R15 = indocyanine green 15-minute retention rate; TBIL = total bilirubin; WBC = white blood cell count.

**Figure 3.**
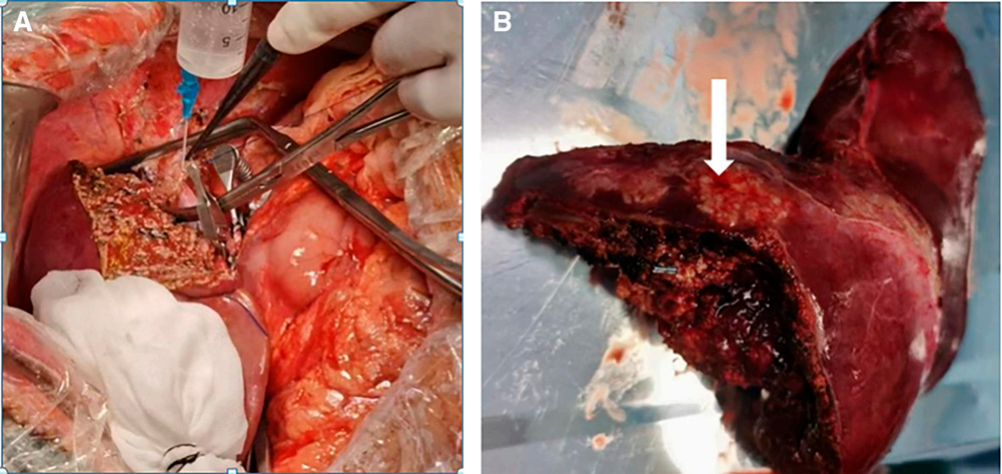
(**A**) The broken end of the right hepatic vein is anastomosed with the broken end of the middle hepatic vein for repair and molding. (**B**) Surgical specimen and alveolar echinococcosis lesion (white arrow).

The operation of semi-ex vivo liver resection autologous liver transplantation (second hepatic portal reconstruction, posterior inferior vena cava repair, and hepatic artery repair) biliary-intestinal anastomosis was performed under general anesthesia on March 10, 2022. During the operation, we found that the sizes of two echinococcus lesions were approximately 7.0 × 4.0 × 3.0 and 6.0 × 3.0 × 3.0 cm, occupying hepatic SI and SIV segments and hepatic SVII and SVIII segments, respectively, with white, hard masses and an uneven surface. The echinococcus lesions of the hepatic SVII and SVIII segments invaded the second hepatic portal and encircled the right hepatic vein and middle hepatic vein; the echinococcus lesions of the hepatic SI and SIV segments invaded the first hepatic portal and the lesions encircled the left branch of the portal vein, left hepatic artery, and left hepatic duct. Splitting the liver along the middle hepatic vein revealed that the echinococcus lesions seriously invaded the posterior inferior hepatic vena cava, right hepatic vein, and middle hepatic vein. Therefore, after resection of the left half of the liver, caudate lobe, and hepatic SVII and SVIII segments of echinococcosis, the suprahepatic inferior vena cava, infrahepatic inferior vena cava, right branch of the portal vein, right hepatic artery, and right hepatic duct were blocked and total hepatic block was performed once, during which block ice bags were applied to the residual liver throughout and the broken end of the right hepatic vein was anastomosed with the broken end of the middle hepatic vein for repair and molding (Figure 3A). The posterior hepatic inferior vena cava and second hepatic portal were repaired and shaped, and the SVI segment of the bile duct was anastomosed with jejunum. A surgical specimen from the patient is shown in Figure 3B. The block was performed once for 39 minutes. Intraoperative bleeding was 150 mL and no intraoperative blood transfusion was performed.

**Figure 4.**
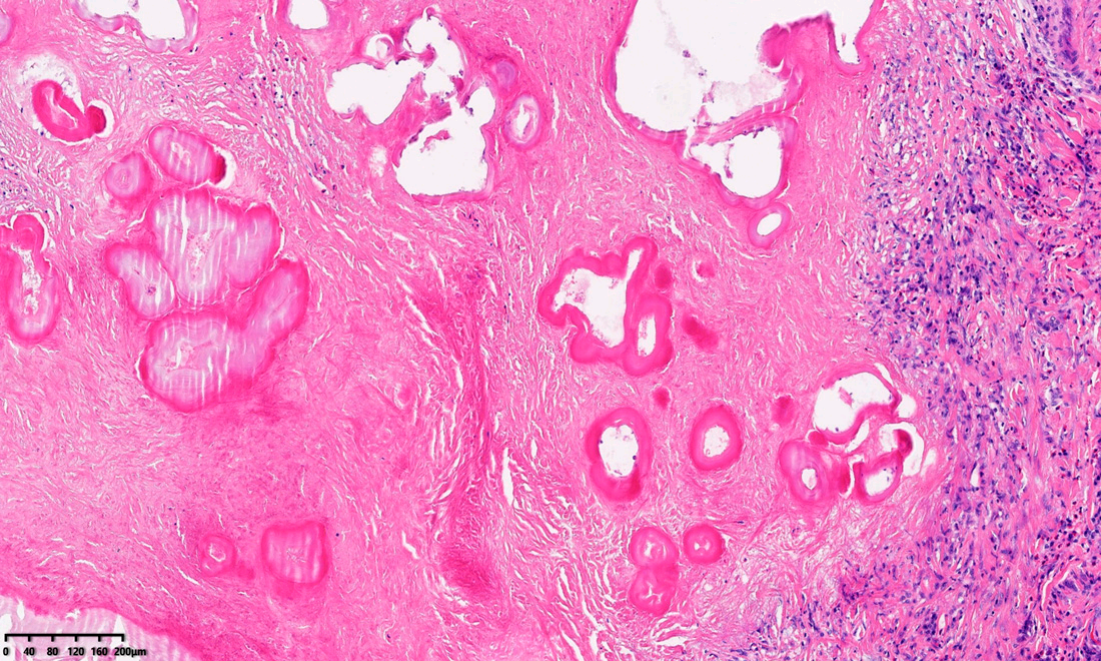
Hepatic alveolar echinococcosis infection.

**Figure 5.**
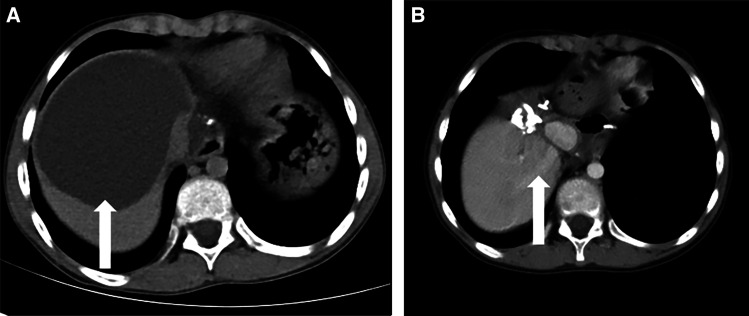
(**A**) Encapsulated fluid (white arrow). (**B**) Ten months after surgery, the effusion was absorbed and the reconstructed right hepatic vein was well filled (white arrow).

The patient recovered well after the operation. Postoperative pathological examination confirmed AE (Figure 4). An angiogram was performed 12 days after the operation, indicating good blood flow in the hepatic vein reconstruction and filling of the right hepatic artery. The patient was discharged successfully on postoperative day 14 and continued to take albendazole orally after discharge. Three months after the operation, she came to the hospital for a reexamination of the abdominal CT scan, which encapsulated fluid in the right lobe of the liver (Figure 5A). The patient was discharged after treatment with puncture tube drainage; 10 months after surgery, reexamination of the abdominal CT scan showed the fluid in the right lobe of the liver had been absorbed and the reconstructed right hepatic vein was well filled (Figure 5B). The patient was advised to continue taking oral albendazole and to follow up regularly.

## DISCUSSION

Alveolar echinococcosis is a chronic disease that is mostly contracted in childhood and is easily overlooked because of the absence of any symptoms in the early stages. Children in areas where echinococcosis is endemic live with poor sanitary conditions, poor economic conditions, and poor self-hygiene habits. These increase the chances of parasitic infection; thus, children are the most representative patients of early echinococcosis.^5^

Semi-ex vivo hepatectomy is a modification of ex-vivo liver resection, in which the main hepatic vein and short hepatic vein, or the superior and inferior hepatic vena cava, are cut off and the liver is turned outside the body for low-temperature perfusion before focal excision.^6^ This operation is mainly used to treat hepatocellular carcinoma, metastatic liver cancer, hepatic AE, and so forth.^7^ It is more frequently used in adult patients and rarely used in pediatric patients. In this case, the lesions of hepatic AE were located in the hepatic segments SI, SIV, SVII, and SVIII near the first and second hepatic portal, and the lesions invaded the vessels surrounding the first and second hepatic portal, which are the right hepatic vein, middle hepatic vein, posterior inferior hepatic vena cava, and part of the first hepatic Glisson capsule, in accordance with indications for semi-ex vivo hepatectomy combined with autologous liver transplantation proposed by Shi and Kang.^6^ Therefore, to achieve R0 resection, we chose semi-ex vivo hepatectomy combined with autologous liver transplantation because it provides sufficient time to complete complex resection and revascularization in a bloodless environment, thus reducing the risk of bleeding.^8^ In addition, Govil^9^ proposed that the liver can safely tolerate ischemia for about 60–90 minutes at room temperature using the conventional Pringle inflow hepatic flow blocking method and that if ex-vivo liver resection is performed, cryoperfusion treatment is required to prolong the period of no liver but should not exceed 2 hours. In this case, the intraoperative period of the child’s liver was 39 minutes; that is, the cold ischemia time was much less than 2 hours, and the intraoperative bleeding was 150 mL. Ice packs were used throughout the whole process to chill the residual liver and reduce the residual liver metabolism, and therefore perfusion treatment was not performed during the operation.

Wang et al.^10^ found that ex vivo hepatectomy combined with autologous liver transplantation is increasingly used in the treatment of hepatic AE and has a good postoperative prognosis. With the improvement of surgical skills, semi-ex vivo liver resection combined with autologous liver transplantation has become available, but preoperative assessment of the patient’s liver reserve function and general condition should be preferred, followed by the improvement of CT three-dimensional reconstruction to clarify the relationship between the alveolar echinococcosis lesions and hepatic vessels and bile ducts.^11^ In addition, intraoperative ultrasound-assisted devices are feasible to further clarify the relationship between the lesion and the blood vessels during surgery, thus helping clinicians to choose the best surgical approach. Compared with adults, for children the duration of the liver-free period should be strictly controlled, so as to reduce postoperative ischemia-reperfusion injury and accelerate the recovery of the child after surgery.^12^ In this case, we used semi-ex vivo hepatectomy with autologous liver transplantation to treat hepatic AE in children. A detailed and thorough surgical plan was formulated before surgery. Intraoperatively, normal liver tissue was preserved as much as possible. The duration of the liver-free period and the amount of bleeding were strictly controlled. Postoperative changes in the patient’s condition and prevention of postoperative complications were closely observed, and the patient was followed up regularly. At present, the patient has no recurrence of AE and lives a normal life. Therefore, we believe that the efficacy of this procedure for treating hepatic AE in children is feasible, but more clinical studies are still needed to support it.

In conclusion, hepatic AE is extremely harmful to children. Early screening, diagnosis, and treatment should be achieved. For some patients with hepatic AE who cannot be cured by conventional liver lobectomy, semi-ex vivo liver resection and autologous liver transplantation may provide a radical treatment chance other than liver transplantation, with good social and economic benefits.
